# SU-Net: pose estimation network for non-cooperative spacecraft on-orbit

**DOI:** 10.1038/s41598-023-38974-1

**Published:** 2023-07-21

**Authors:** Hu Gao, Zhihui Li, Ning Wang, Jingfan Yang, Depeng Dang

**Affiliations:** 1 School of Artificial Intelligence, Beijing Normal University, Beijing, 100000 China; 2 China Aerodynamics Research and Development Centre, Mianyang, 621000 China

**Keywords:** Aerospace engineering, Computer science, Information technology

## Abstract

The estimation of spacecraft pose is crucial in numerous space missions, including rendezvous and docking, debris removal, and on-orbit maintenance. Estimating the pose of space objects is significantly more challenging than that of objects on Earth, primarily due to the widely varying lighting conditions, low resolution, and limited amount of data available in space images. Our main proposal is a new deep learning neural network architecture, which can effectively extract orbiting spacecraft features from images captured by inverse synthetic aperture radar (ISAR) for pose estimation of non-cooperative on orbit spacecraft. Specifically, our model enhances spacecraft imaging by improving image contrast, reducing noise, and using transfer learning to mitigate data sparsity issues via a pre-trained model. To address sparse features in spacecraft imaging, we propose a dense residual U-Net network that employs dense residual block to reduce feature loss during downsampling. Additionally, we introduce a multi-head self-attention block to capture more global information and improve the model’s accuracy. The resulting tightly interlinked architecture, named as SU-Net, delivers strong performance gains on pose estimation by spacecraft ISAR imaging. Experimental results show that we achieve the state of the art results, and the absolute error of our model is 0.128$^{\circ }$ to 0.4491$^{\circ }$, the mean error is about 0.282$^{\circ }$, and the standard deviation is about 0.065$^{\circ }$. The code are released at https://github.com/Tombs98/SU-Net.

## Introduction

With the continuous advancement of space technology and the increasing diversity of space missions, on-orbit servicing has emerged as a crucial approach to ensuring the stable operation of spacecraft in complex space environments. In such scenarios, precise estimation of the pose of a non-cooperative spacecraft plays a vital role in facilitating close-proximity operations, including debris removal, target tracking, and monitoring. A precise pose estimation model not only saves time but also enables relevant personnel to obtain pose data in a timely manner. This is particularly crucial for research involving subsequent docking, maintenance operations, determining the orbital decay of on-orbit spacecraft, calculating flight pose angles, and measuring the angle between solar panels and two hulls. Furthermore, it serves as a prerequisite for calculating aerodynamics during the reentry processes of large spacecraft from rails in subsequent operations.

**Figure 1:**
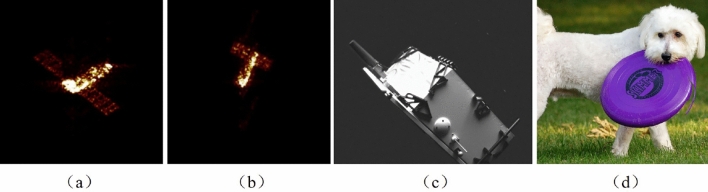
Image used for pose estimation. Figure (**a**) and (**b**) shows the spacecraft ISAR imaging, with (**a**) exhibiting relatively comprehensive and clear results, while (**b**) showcases poorer image quality. Figure (**c**) shows the image taken via satellite. Figure (**d**) shows the image on Earth.

Due to the absence of cooperation information, non-cooperative spacecraft on-orbit cannot directly utilize sensors to obtain attitude data. Currently, the tasks of non-cooperative spacecraft on-orbit can be broadly categorized into two types^1^. The first involves capturing images by leveraging other satellites dedicated to space photography^2^. The second method entails imaging the spacecraft in orbit using ground-based techniques such as inverse synthetic aperture radar (ISAR)^3,4^, eliminating the need for a specialized satellite and relying solely on radar imaging from the ground. As shown in Fig. 1, pose estimation using ISAR imaging differs significantly from target pose estimation on Earth and satellite imaging. The challenging space conditions and the significant distance further exacerbate the difficulties associated with ISAR imaging. Consequently, adequate light dispersion is impeded, resulting in extremely high imaging contrast and occasional incompleteness in the captured images. Additionally, technical equipment limitations contribute to relatively low image resolution. As a result, the mainstream pose estimation methods currently employed on Earth, such as HRNet^5^, MIPNet^6^, Transpose^7^, and AggPose^8^, cannot be directly applied to pose estimation of spacecraft on-orbit.

In the past few years, there has been growing interest in using stereoscopic cameras to analyze images of target satellite engines and visual measurement tools to calculate their relative pose for known targets^9–12^. However, these methods often rely on simplified geometries, involve a significant number of formula equation solutions, and require a long operation time. For instance, one approach involves using an axial analysis method based on morphological linear mechanism corrosion to extract features from low-resolution spacecraft images and running a matching scheme, such as RANSAC with PnP (Perspective-n-Point) for model evaluation or SoftPOSIT^13^. The results of feature extraction significantly affect subsequent target pose estimation.

The rapid development of artificial intelligence has led to the implementation of convolutional neural networks (CNNs) for pose estimation of non-cooperative spacecraft on-orbit, which has become an attractive solution in recent years^2,12,14–18^. However, the pose estimation of non-cooperative spacecraft on-orbit based on deep learning mostly uses simulation data or images taken by other satellites as model training sets to train models. Although models trained with simulation data demonstrate effectiveness during testing, they encounter numerous challenges when applied to real-world scenarios. Acquiring images of the target to be estimated by a satellite is expensive and data availability is limited.

Based on the information presented, a natural question that comes to mind is whether it is feasible to utilize more easily accessible ISAR imaging of the spacecraft for pose estimation? To achieve this objective, we analyze the characteristics of spacecraft ISAR imaging, including low resolution, blurred contour boundaries, incomplete panel imaging, relatively simple image semantics, and fixed structure, as shown in Fig. 1a and b. Subsequently, we propose a pose estimation network specifically designed for on-orbit ISAR imaging of non-cooperative spacecraft, named SU-Net, with several key components. 1). An image enhancement algorithm is applied to boost image contrast and reduce noise. 2). A transfer learning approach is adopted to overcome the problem of limited spacecraft ISAR imaging data and facilitate model training using pre-trained models. 3). A dense residual Block (DRB) is employed to allow for the reuse of feature maps from previous layers and helps to maintain information flow and multi-scale fusion through the network. This results in improved feature representation, alleviates the issue of sparse image features, and overcome the problem of feature loss caused by downsampling, making it easier for the model to capture important features during training. 4). A multi-head self-attention block (MHSAB) is integrated to capture global information, enabling the model to represent richer semantic information. Notably, this paper is the first to present an pose estimation network for spacecraft ISAR imaging to the best of my knowledge. The contributions of this paper are summarized as follows:

1. A novel pose estimation network architecture for spacecraft ISAR imaging capable capturing contextually-enriched information and spatially accurate details, enabling efficient and accurate pose estimation.
2. A contrast limited adaptive histogram equalization and transfer learning that not only enhances image contrast and reduces noise, but also addresses the challenge of model training with limited spacecraft ISAR imaging data.
3. A dense residual block that facilitate information flow and multi-scale fusion across the network, mitigating the issue of sparse image features and compensating for feature loss caused by subsampling.
4. A multi-head self-attention block to capture more global information.
5. The effectiveness of the SU-Net is demonstrated by setting a new state-of-the-art on the datasets of spacecraft ISAR imaging for pose estimation. Furthermore, detailed ablations, qualitative results, and generalization tests are provided to support our findings.

## Related work

### Pose estimation

Pose estimation involves determining pose data from an image or video. In this paper, we introduce object pose estimation on Earth and spacecraft pose estimation respectively.

#### Object pose estimation on Earth

The pose estimation methods of objects on Earth can be divided into two categories: end to end and two-stage. (1) End-to-end method is a technique that can convert a two-dimensional image or its feature representation into a six-dimensional output space without relying on a geometric solver. Traditionally, the approach involved manually matching the imaging region with database data^19,20^. With the data-driven methods such as CNNs^21–25^ have become the preferred approach for pose estimation due to their exceptional performance. These methods directly regress the final pose result or first classify before regression. (2) Two-stage pose estimation methods typically involve first estimating the 2D image projection of the 3D object keypoints, followed by applying a Perspective-n-Point (PnP) algorithm to the set of 2D-3D correspondences to retrieve the pose. Currently, many works^22,26–28^ are based on CNNs to regress key-points by employing heatmaps or by designing 2D keypoints that can detect vertices and check the boundary box of 3D objects, in order to improve the accuracy and efficiency of the pose estimation process.

#### Spacecraft pose estimation

Due to the complex space conditions, the pose estimation for non-cooperative spacecraft on-orbit is a different task compared to object pose estimation on Earth. The traditional pose estimation of spacecraft is mainly based on visual computing tools^4^, model matching^14,29–31^ and texture feature extraction^3^. With the advancement of transfer learning, the constraint of data volume for model training has been lifted. Using the shared weight transfer learning method, researchers^2,16–18,32,33^ leveraged pre-trained models to initialize the weights of their own models. They then fine-tuned the models on their own datasets to either regress the pose angle or classify a discrete pose of the target spacecraft. In order to enhance the accuracy of spacecraft pose estimation, certain individuals may also devise novel model structures or loss functions^1,34,35^. Although satisfactory results have been obtained when applying these methods to simulation data or images captured by other satellites (such as speedplus dataset^36,37^), their application to spacecraft ISAR imaging data yields poor pose estimation results.

### U-Net

A U-Net network model was originally proposed for semantic segmentation, consisting of a shrink path for context information and an expansion path for precise positioning. To enhance its effectiveness, subsequent researchers have improved the model, such as^38–41^. While the U-Net network is predominantly utilized for semantic segmentation of medical images, such as X-rays, CT scans, and MRI scans, its application to pose estimation of spacecraft ISAR imaging remains unexplored. In this paper, we analyze the differences between spacecraft ISAR images and medical images, such as their simple semantics, relatively fixed structures, and unclear boundary contours. Based on this analysis, we propose the SU-Net architecture, which utilizes the U-Net architecture to achieve spacecraft pose estimation.

### Dense connection and residual connection

Huang et al.^42^ presents a dense convolutional network that connects each layer to every other layer in a feedforward manner, allowing for direct access to gradients from the loss function and input signal. This approach overcomes the issue of gradient disappearance and promotes feature propagation and reuse. To address the difficulty of training deep networks, He et al.^43^ introduced the residual network, which not only prevents gradient vanishing but also reduces model complexity to mitigate overfitting. Subsequently, several papers such as^44–48^ proposed different residual network structures tailored to specific tasks. In particular, Wang et al.^46^ combined residual and dense connections.

### Attention

Due to the attention mechanism’s ability to capture global information, an increasing number of visual tasks now incorporate attention, including image classification^49–51^, segmentation^52,53^ and detection^51,54^. Xu et al.^55^ successfully utilized visual transformers to estimate human poses and achieved impressive results, however, the utilization of this model has also led to an increase in its network system complexity.

## Method

**Figure 2:**
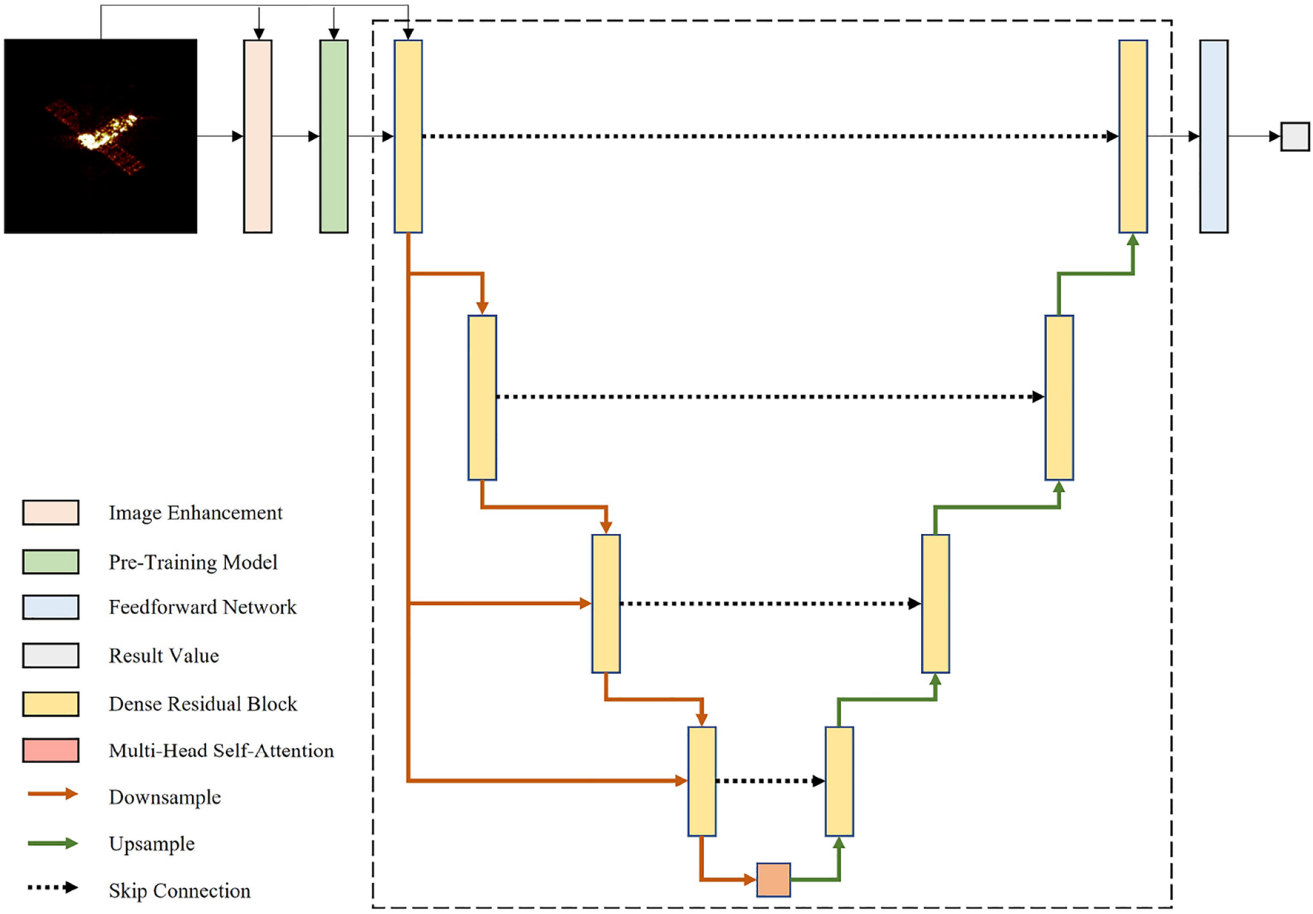
Architecture of SU-Net for spacecraft pose estimation by ISAR imaging. The meaning of all types of signs is marked at the bottom left corner.

The framework proposed for spacecraft ISAR imaging pose estimation, as depicted in Fig. 2, involves four steps: (a) Image enhancement (IE) to improve image contrast and reduce noise, (b) Extracting shallow features and overcoming the limitations of the dataset size by employing a pre-trained model (PM), (c) Extracting deep features by dense residual U-Net (DR-U-Net), and (d) Regressing the pose result. It is worth noting that spacecraft ISAR imaging features are sparse. To minimize feature loss during image preprocessing and pre-training, residual connections have been introduced to prevent both feature loss and model degradation.

**Overall pipeline** Given a spacecraft ISAR image $\textbf{X} \in \mathbb R^{H \times W \times 3}$, SU-Net first applies a contrast limited adaptive histogram equalization ( CLAHE ) to enhance image quality to obtain ${{\bf X}_{\bf e}} \in \mathbb R^{H \times W \times 3}$. Next, the origin image $\textbf{X}$ and enhanced image ${{\bf X}_{\bf e}}$ pass through the pre-training model to extract shallow feature maps ${{\bf X}_{\bf r}} \in \mathbb R^{H \times W \times C}$. Then these shallow features ${{\bf X}_{\bf r}}$, ${{\bf X}_{\bf e}}$ and $\textbf{X}$ pass through $4-level$ encoder–decoder and one multi-head attention middle block, yielding deep features ${{\bf X}_{\bf d}} \in \mathbb R^{H \times W \times C}$. Finally, we apply a convolution and a feed-forward network to deep features ${{\bf X}_{\bf d}}$ and generate the pose information.

### Image enhancement (IE)

The raw images obtained from spacecraft ISAR imaging exhibit low resolution, fuzzy boundaries, and high levels of noise. When these images are fed directly into a neural network, the results are often suboptimal because the network captures a plethora of irrelevant features. To improve the outcomes, we use the CLAHE^56^ to filter out noise and enhance their contrast and clarity before feeding them into the neural network.

**Figure 3:**
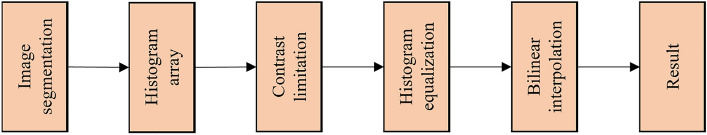
The Steps of CLAHE.

As shown in Fig. 3, firstly, the image is padded to ensure that it can be segmented into non-overlapping sub-blocks of equal size. Next, a histogram array is generated for each sub-block. The contrast of the image is then limited and the extra pixels are redistributed using a pixel reallocation method. Afterwards, a histogram equalization mapping function is generated for each sub-block based on the restricted histogram. Finally, the output image is obtained using bilinear interpolation, which involves calculating the value of each pixel based on its position in relation to the neighboring pixels. To preprocess the ISAR imaging data of each spacecraft, we start by resizing its feature tensor $\textbf{X}$ to $256 \times 256$ and then apply CLAHE processing to enhance its contrast and improve the image quality. The resulting feature is denoted as ${{\bf X}_{\bf e}}$ as follows:

1 $$\begin{aligned} {{\bf X}_{\bf e}} = CLAHE(\textbf{X}) \end{aligned}$$

### Pretraining model (PM)

Data-driven methods usually require a large amount of data to train the model so that it can capture the characteristics of the data and establish the mapping relationship between the input and output. However, the data of spacecraft ISAR images is small, and the data features are sparse, which makes the model difficult to train. To this end, we introduce transfer learning, which uses the pre-trained model to extract image shallow features and initialize the model shallow parameters to speed up the training process. Although most of the pre-trained models^43,57,58^ are based on the ImageNet dataset^59^ and there are large differences between images and spacecraft ISAR imaging, they are all composed of color images of a primary object^60^ and adjusting the original weights after training on a new dataset can improve the performance of the model. At the same time, in order to reduce the loss characteristics of image preprocessing, the original spacecraft ISAR imaging features $\textbf{X}$ is added to the enhanced features ${{\bf X}_{\bf e}}$, and input into the ResNet34 pre-trained model to obtain the shallow features ${{\bf X}_{\bf r}}$, as follows:

2 $$\begin{aligned} {{\bf X}_{\bf r}} = ResNet(\textbf{X}+ {{\bf X}_{\bf e}}) \end{aligned}$$

### Dense residual U-Net (DR-U-Net)

The DR-U-Net is built upon a U-Net like architecture, as shown inside the dashed box in Fig. 2. As is apparent from the figure, in contrast to the traditional U-Net network, we have introduced two key components: (**a**) the dense residual block (DRB) and (**b**) the multi-head self-attention block (MHSAB). We add $\textbf{X}, {{\bf X}_{\bf e}}$ and ${{\bf X}_{\bf r}}$, and feed them into the DR-U-Net. Formally, let ${{\bf XE}_{\bf i}}$ be the output features at level $i (i = 1, 2, 3, 4)$ in the encoder part. At each level *i* in the decoder part, the input features ${{\bf XD}_{\bf i}}$ is given as:

3 $$\begin{aligned} & {{\bf XD}_{\bf i}} = {{\bf XE}_{\bf i}} + UP( {{\bf XD}_{{\bf i}+{\bf 1}}})\\& {{\bf XD}_{\bf 4}} = {{\bf XE}_{\bf 4}} + UP(MHSAB( {{\bf XE}_{\bf 4}}))\\& {{\bf XE}_{\bf i}} = DRB(DOWN( {{\bf XE}_{\bf 1}}) + {{\bf XE}_{{\bf i-1}}})\\& {{\bf XE}_{\bf 1}} = DRB(\textbf{X} + {{\bf X}_{\bf e}} + {{\bf X}_{\bf r}})\\& {{\bf XE}_{\bf 0}} = None \end{aligned}$$

where $UP(\cdot )$ denotes the upsample operation, $MHSAB(\cdot )$ denotes the multi-head self-attention block, and $DRB(\cdot )$ is the dense residual block. Finally, the depth features ${{\bf X}_{\bf d}}$ are obtained through DR-U-Net, as demonstrated below:

4 $$\begin{aligned} {{\bf X}_{\bf d}} = DRB( {{\bf XD}_{\bf 1}}) \end{aligned}$$

It’s worth noting that in the Fig. 2, we can see that the output of the first encoding block is passed to all the subsequent encoder blocks. This approach is beneficial for reducing feature loss and improving the overall performance of the model.

**Figure 4:**
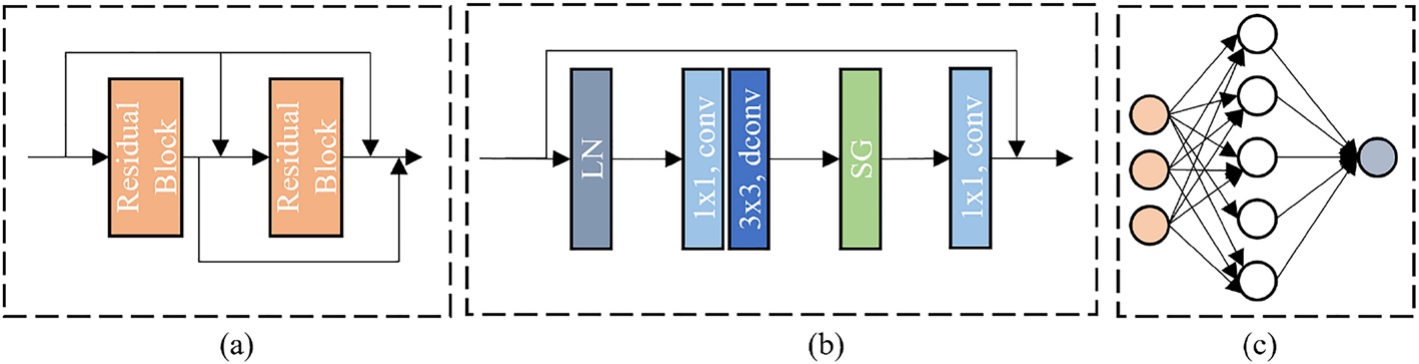
(**a**) DRB. (**b**) The residual block in the DRB. (**c**) The feedforward network to generate the pose result.

#### Dense residual block (DRB)

As shown in Fig. 4a, the dense residual blocks (DRB) is a dense connection with multi residual blocks (RB). And each RB contains Layer Normalization, Convolution, Simple Gate and residual connection as shown in Fig. 4b. Given the input feature $\textbf{F}$, we extract features ${{\bf F}_{\bf d}}$ by DRB as:

5 $$\begin{aligned} & {{\bf F}_{\bf d}} = RB(RB(\textbf{F})+\textbf{F}) +RB(\textbf{F}) + \textbf{F}\\&RB(\textbf{F}) = \textbf{F} + C_1(SG(C_3(C_1(LN(\textbf{F})))))\\&SG(\textbf{F}) = { {\bf F}_{{\bf f1}}} \times {{\bf F}_{{\bf f2}}} \end{aligned}$$

where $C_1$ is the $1 \times 1$ convolution, $C_3$ is the $3 \times 3$ depth-wise convolution, LN denotes Layer Normalization, and ${{\bf F}_{{\bf f1}}, {\bf F}_{{\bf f2}}}$ are obtained by dividing $\textbf{F}$ into channel dimensions. This design offers three advantages. Firstly, it incorporates a residual connection that enables the pose estimation module to aggregate features from various levels through a short identity-based connection between different blocks. Secondly, it includes a dense connection that reduces feature loss during transfer, and mitigates the difficulty of training the model due to the sparsity of spacecraft ISAR imaging features. Furthermore, to reduce system complexity and resource consumption, we replaced the original convolution operation with a depthwise separable convolution and introduced a simple gating mechanism that uses multiplication instead of a nonlinear activation function.

**Figure 5:**
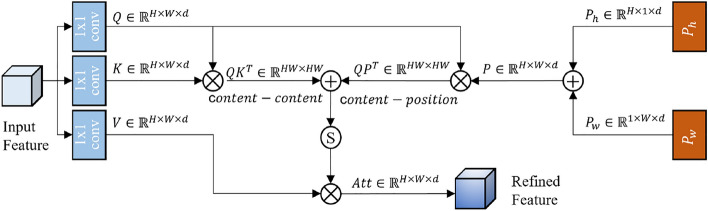
The architecture of Multi-Head Self-Attention Block used in the SU-Net. We show the only one head for simplicity.

#### Multi-head self-attention block (MHSAB)

The transformer model^61^ has become a favored tool for vision tasks because of its ability to capture global information, which has led to its growing usage in recent times. However, its computation on a global scale results in a quadratic complexity in relation to the number of tokens, as demonstrated by Eq. (6), where *MSA*, *h*, *wandC* represent multi-head self-attention, the image height, width and channel respectively. This limitation makes it unsuitable for representing high-resolution images.

6 $$\begin{aligned} \Omega _{MSA} = 4hwC^2 + 2(hw)^2C \end{aligned}$$

To address this problem, we suggest using a multi-head self-attention block (MHSAB) as a connector between the encoder and decoder, as illustrated in Fig. 5. The MHSAB utilizes global self-attention to process and merge feature map data from the encoder’s last layer. This technique is especially effective when dealing with large images because convolution downsamples space, while attention can effectively handle smaller resolutions, resulting in improved performance.

From the last layer of the encoder output $XE_4$, our MHSAB first apply a $1 \times 1$ pointwise convolution to generate the *query*, *key* and *value* matrices $\textbf{Q} \in \mathbb R^{H \times W \times d}, \textbf{K} \in \mathbb R^{H \times W \times d}$ and $\textbf{V} \in \mathbb R^{H \times W \times d}$ as follows:

7 $$\begin{aligned} &\textbf{Q} = C_1( {{\bf XE}_{\bf 4}}) \\&\textbf{K} = C_1({{\bf XE}_{\bf 4}}) \\&\textbf{V} = C_1( {{\bf XE}_{\bf 4}}) \end{aligned}$$

To effectively correlate information between location-aware objects, our attention mechanism considers both content information and the relative distance between different location features. We represent the relative distance position encoding for height and width as ${{\bf P}_{\bf h}} \in \mathbb R^{H \times 1\times d}$ and ${{\bf P}_{\bf w}} \in \mathbb R^{1 \times W\times d}$ respectively. The attention logits are calculated as ${{\bf QK}^{\bf T}} + {{\bf QP}^{\bf T}}$, where ${{\bf QK}^{\bf T}}$ represents the content-content interactions and ${{\bf QP}^{\bf T}}$ represents the content-position interactions.

Next, the attention matrix is thus computed by the self-attention mechanism as:

8 $$\begin{aligned} Attention(\textbf{Q,K,P,V}) = SoftMax( {{\bf QK}^{\bf T}+{\bf QP}^{\bf T}})\textbf{V} \end{aligned}$$

By incorporating the self-attention mechanism into our model, we can calculate the correlation between different positions in the sequence and assign weights to each position. This enables us to globally focus on the sequence and dynamically model long-distance dependencies. Moreover, it helps reduce parameter coupling, thereby enhancing the expressiveness and performance of the model. Finally, we reshape the attention matrix back to its original dimensions of $\mathbb {R}^{H \times W \times d}$. The resulting output is then added to $XE_4$ and passed to the decoder. By utilizing this approach, we can gather a greater amount of information and improve the overall performance of the model, as evidenced by the following experiment.

### Feed-forward network

The final pose result can be obtained by regressing the deep features ${{\bf X}_{\bf d}}$ obtained by DR-U-Net through a feed-forward network, as illustrated in Fig. 2. The feed-forward network, depicted in Fig. 4c), consists of layers of neurons that are connected in a sequential manner. Each neuron is connected only to the neurons of the preceding layer, and it receives the output of that layer while providing its output to the next layer. The process is as follows :

9 $$\begin{aligned} \hat{y} = {{\bf W}_{\bf 1}({\bf W}_{\bf 2}({\bf X}_{\bf d}))} \end{aligned}$$

where ${{\bf W}_{\bf i}}$ is the weight vector and $\hat{y}$ denotes the predicted pose result value.

### Loss function

To train the model suggested in this paper, we utilized three distinct loss functions, namely L1 loss (also known as mean absolute error or MAE), L2 loss (also known as mean squared error or MSE), and Huber loss. Among these, L1 loss (Eq. 10) represents the average absolute difference between the *n* predicted $\hat{y}$ and *n* target *y* values. This loss function quantifies the average error magnitude of a set of predictions, without considering their direction.

10 $$\begin{aligned} MAE = \frac{\sum _{i=1}^{n} |y_i - \hat{y_i} |}{n} \end{aligned}$$

The L2 loss function, shown in Eq. (11), is widely utilized for regression tasks as it calculates the sum of the squared differences between the predicted and target values. This loss function is highly preferred due to its common usage.

11 $$\begin{aligned} MSE = \frac{\sum _{i=1}^{n} (y_i - \hat{y_i})^2}{n} \end{aligned}$$

In spacecraft ISAR imaging, the training data contains outliers that are individual in nature, owing to the limitations of ISAR equipment. The MSE loss function assigns greater weightage to these outliers, compared to the MAE loss function. By minimizing the impact of a single outlier case, the model inadvertently compromises its performance on other commonly occurring examples, resulting in an overall degradation of performance.

To overcome this limitation, the huber loss function can be used, which is less sensitive to outliers in the data and can be differentiated at zero (as depicted in Eq. 12). This function essentially computes the absolute error, but switches to mean squared error when the error is small. The hyperparameter $\delta$ controls the sensitivity of this error, and is set to 1 in this paper.

12 $$\begin{aligned} L_{\delta }(y,\hat{y}) = {\left\{ \begin{array}{ll} \frac{1}{2} (y-\hat{y})^2 &{} |\text { y }-\hat{y} |\le \delta ,\\ \delta |y-\hat{y} |- \frac{1}{2}\delta ^2&{} \text { otherwise. } \end{array}\right. } \end{aligned}$$

## Experiment

In order to assess the effectiveness of our method, we perform comparative tests, ablation experiments, and analyze the impact of loss on the results. By conducting ablation experiments, we systematically remove certain components of the model and compare the performance differences between different ablation versions and the complete model. This allows us to assess the effectiveness of our proposed DRB and MHSAB, demonstrating their significance in improving the overall model performance. Additionally, we discussed the impact of various loss functions on the accuracy of pose estimation.

### Setup

#### Datasets

**Figure 6:**
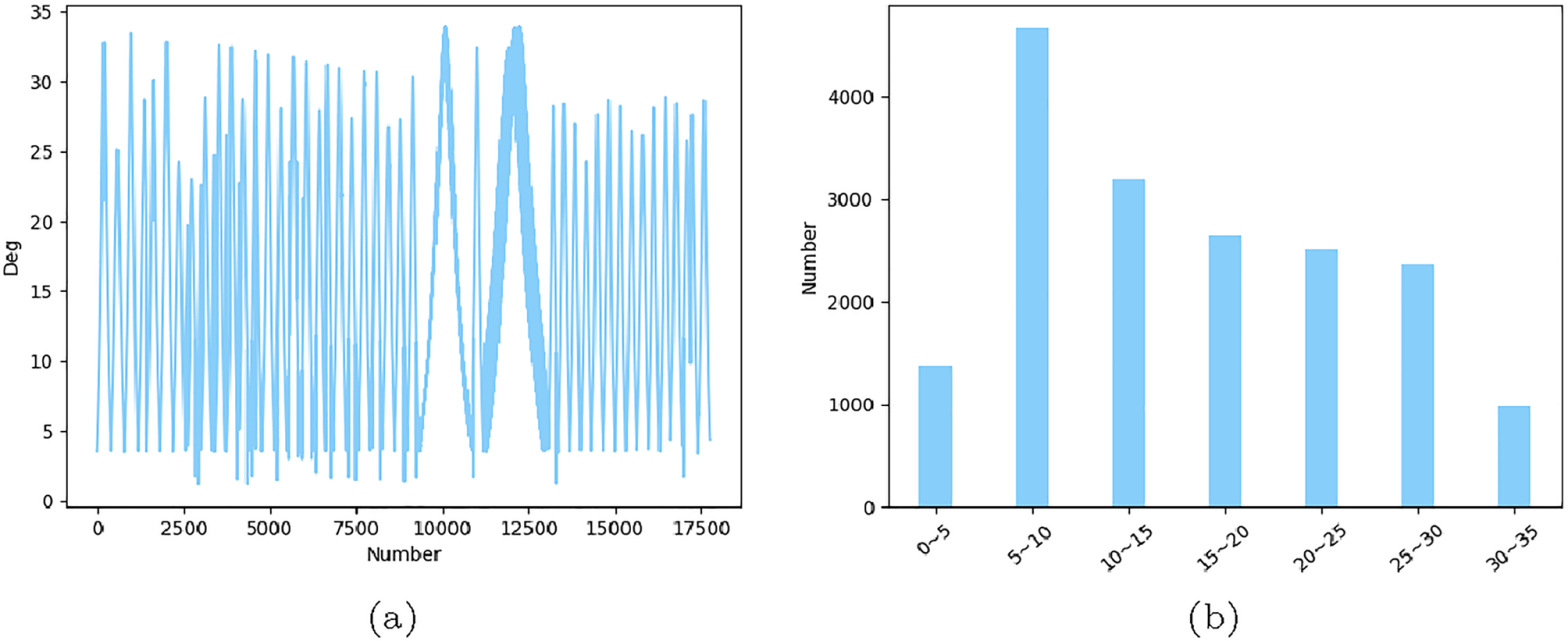
The distribution of dataset. Figure (**a**) shows the pose angle of all images. Figure (**b**) shows the number of pose angel in each interval.

The paper utilizes a data set of 17,744 on-orbit ISAR imaging data from a specific spacecraft. The data set is divided into a training set consisting of $80\%$ of the data and a test set consisting of $20\%$ of the data. The distribution of the data samples can be found in Fig. 6. Notably, the ISAR imaging technology is only able to detect the spacecraft within a limited range, resulting in all images having pose angles between $0^{\circ }$ and $35^{\circ }$, with a significant concentration between $5^{\circ }$ and $10^{\circ }$.

#### Training and metrics

We use Adam optimization with $\beta _{1}=0.9$ and $\beta _{2}=0.999$, a learning rate of 0.0005, and a batch size of 16 to train all models. We apply linear learning rate warmup and decay. The accuracy of pose estimation on our dataset is reported using either minimum absolute error (MinAE), max absolute error (MaxAE), and mean absolute error (MeanAE). The minimum absolute error is the smallest difference between the target value and predicted value of the model when predicting a batch of poses. The mean absolute error is the average error of the model when predicting a batch of poses. Additionally, we calculate the standard deviation (Std) to measure the spread of a set of forecasting errors.

### Comparative tests

To assess the performance of the proposed SU-Net, we conduct a comparative evaluation against the prevailing method for spacecraft pose estimation^1^, which is based on the speedplus^62^ or other satellite image dataset (listed in the second box of the Table 1), as well as against the prevalent method for human pose estimation^5,6,8^ (listed in the third box of the Table 1).

**Table 1:** Compare with the state-of-the-art methods on the dataset used in this paper. In the second box, we list the methods based on speedplus^62^ or other satellite image datasets, including the works by^1,2,17,18,32,33^. In the third box, we include the methods for human pose estimation, such as^5,6,8^. Best are **highlighted** and second are underlined.

Methods	MinAE	MaxAE	MeanAE	Std
2021 Yang(AlexNet)^33^	$0.908^{\circ }$	$1.424^{\circ }$	$0.991^{\circ }$	$0.046^{\circ }$
2021 Yang(ResNet)^33^	$0.534^{\circ }$	$0.884^{\circ }$	$0.623^{\circ }$	$0.037^{\circ }$
2020 Lorenzo^18^	$0.942^{\circ }$	$1.426^{\circ }$	$1.023^{\circ }$	$0.052^{\circ }$
2020 Alexei^32^	$0.914^{\circ }$	$1.343^{\circ }$	$0.980^{\circ }$	$0.041^{\circ }$
2020 Shubham^2^	$0.897^{\circ }$	$1.222^{\circ }$	$0.959^{\circ }$	$0.043^{\circ }$
2020 Xu^17^	$1.803^{\circ }$	$4.051^{\circ }$	$2.161^{\circ }$	$0.640^{\circ }$
2022 Juan^1^	$\underline{0.303^{\circ }}$	$\underline{0.489^{\circ }}$	$\underline{0.401^{\circ }}$	$0.051^{\circ }$
2019 HRNet^5^	$0.583^{\circ }$	$0.699^{\circ }$	$0.661^{\circ }$	$0.034^{\circ }$
2021 MIPNet^6^	$0.576^{\circ }$	$0.661^{\circ }$	$0.646^{\circ }$	$\underline{0.031^{\circ }}$
2022 AggPose^8^	$0.551^{\circ }$	$0.639^{\circ }$	$0.625^{\circ }$	${{\bf 0.024}^{\circ }}$
**SU-Net (our)**	${{\bf 0.128}^{\circ }}$	${{\bf 0.432}^{\circ }}$	${{\bf 0.282}^{\circ }}$	$0.063^{\circ }$

In order to obtain the results of these methods on our dataset. We used the code that their original paper provided to Github. It is worth noting that there are some papers here that do not provide code, but according to the introduction of their papers, just take a typical deep learning model, use the pytorch library, and then fine-tune it. Therefore, we also adopted this method. For the setting of experimental parameters, we all set the same. Among them, using the heat map regression model, we adopt the direct regression method, but this does not affect the comparison of the final method.

In addition, in order to enhance our persuasive, we added experiments on the speedplus dataset. We use the pose score which is based on the combination of the position score $Sets_{sp}$ and the orientation score $Sets_{so}$ to evaluate the performance. $Sets_{sp}$ and $Sets_{so}$ are given from Eqs. (13, 14) respectively, where *y* is the ground-truth and $\hat{y}$ is the estimated. As shown in Table 2, and we also achieved considerable results, improving both on lightbox and sunlamp compared to the baseline of the speedplus dataset paper.

13 $$\begin{aligned} Sets_{sp}= &\; {} Min(0, \frac{|y-\hat{y} |_2}{|y |_2}) \end{aligned}$$

14 $$\begin{aligned} Sets_{so}= &\; {} 2 \cdot arccos(|\left\langle y, \hat{y} \right\rangle |) \end{aligned}$$

According to the Table 1, our method better captures the spacecraft ISAR imaging characteristics and yields more accurate pose estimation results. Our method outperforms the SpeedPlus-based spacecraft estimation method, reducing the mean absolute error by $0.119^\circ$. Similarly, compared to the method based on human pose estimation, our method reduces the mean absolute error by $0.343^\circ$.

**Table 2:** Compare with the state-of-the-art methods for human pose estimation on the speedplus dataset.

Methods	$Lightbox_{sp}$	$Lightbox_{so}$	$Lightbox_s$	$Sunlamp_{sp}$	$Sunlamp_{so}$	$Sunlamp_s$
Baseline	0.3686	2.2038	2.5724	0.3736	2.2002	2.5738
2019 HRNet	0.2592	0.6812	0.9404	0.2207	0.6193	0.8400
2021 MIPNet	**0.1271**	0.5427	0.6698	**0.1199**	0.5351	0.6550
**Su-Net(our)**	0.2001	**0.3549**	**0.5550**	0.2074	**0.4059**	**0.6133**

**Figure 7:**
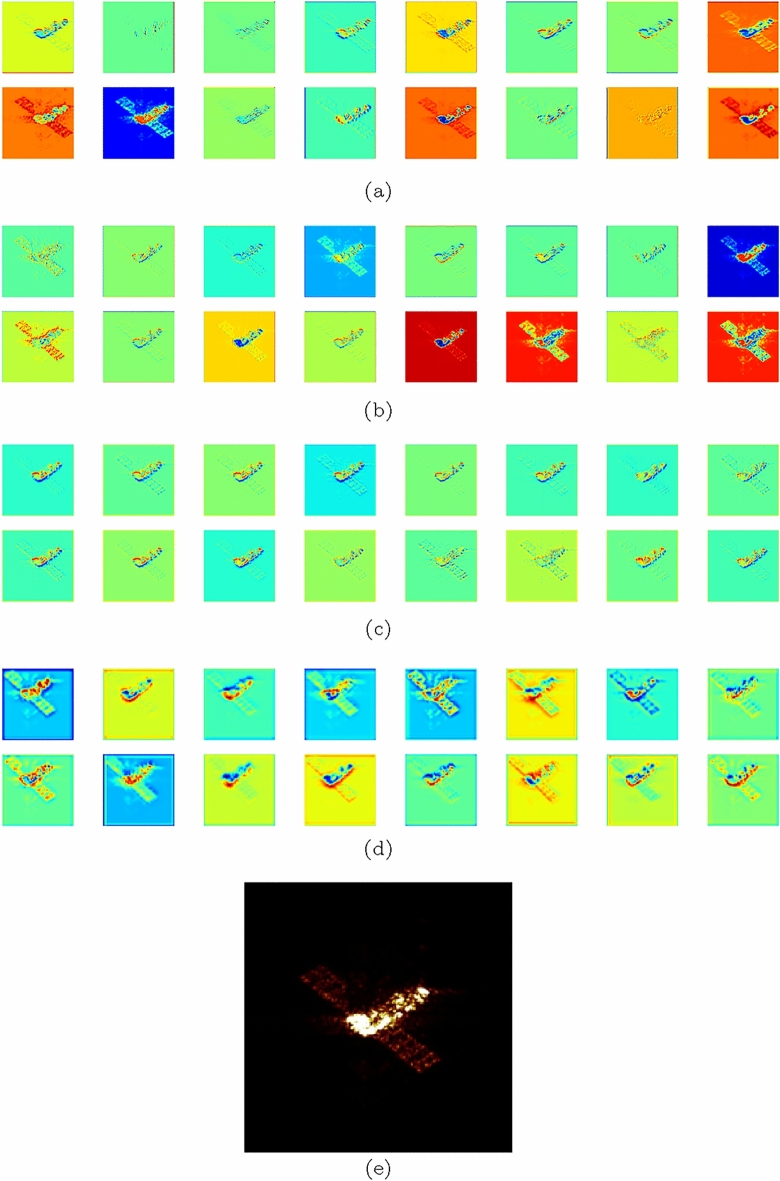
Feature map visualization. Figure **a** is ours. Figure **b**–**d** are produced by the models proposed by^17,18,33^ respectively. Figure **e** is the image which input the model.

In Fig. 7, we present a visualization of the feature maps extracted from^17,18,33^ and ours. Based on the analysis of Fig. 7a, we can conclude that our model is more effective in capturing the ISAR imaging features of the spacecraft, leading to more accurate pose estimation. However, in some cases, as shown in Fig. 7b, although rich feature information can be extracted, there may be a significant amount of noise present. Additionally, as seen in Fig. 7c and d, the extracted features may be incomplete or overly noisy.

### Ablation experiments

Here we present ablation experiments to analyze the contribution of each component of our model, and the results are shown in Table 3.

**IE** Our model yields worse performance and the pose estimation error increase as the image enhancement is removed, which validates the effectiveness of our design.

**PM** It has been demonstrated that incorporating pre-trained models can decrease the reliance on data size and enhance the model’s ability to capture more feature information. Our experiments examined the effects of utilizing a pre-trained model on our results and confirmed that removing the pre-trained model caused our pose estimation error to increase by $0.043^\circ$.

**DRB and MHSAB** We demonstrate the effectiveness of the proposed dense residual block and multi-head self-attention block by replacing or removing them from our final model. Table 3 shows a substantial improve in mean absolute error from $0.282^\circ$ to $0.394^\circ$ when DRB is replaced by the original U-Net convolution, and from $0.282^\circ$ to $0.289^\circ$ when we take out MHSAB.

**Table 3:** Ablation study on individual components of the proposed MSNet.

	SU-Net	- IE	- PM	- DRB	- MHSAMB
MinAE	$0.128^\circ$	$0.142^\circ$	$0.214^\circ$	$0.372^\circ$	$0.135^\circ$
MeanAE	$0.282^\circ$	$0.287^\circ$	$0.325^\circ$	$0.394^\circ$	$0.289^\circ$

### The influence of loss functions

In this paper, we investigate the impact of various loss functions on the final outcomes, as presented in Table 4. The use of the huber loss function yields smaller minimum absolute error and mean absolute error values compared to those obtained using the MAE and MSE loss functions. However, when the MAE loss function is employed, the standard deviation is the lowest. This is because the MAE loss function is not sensitive to outliers and does not prioritize minimizing individual outliers at the expense of other typical examples. Consequently, the standard deviation of the prediction error for the final model is relatively lower than that of the other two loss functions.

**Table 4:** The influence of different loss functions on the results. Best are **highlighted** and second are underlined.

Loss function	MinAE	MaxAE	MeanAE	Std
MSE loss	$0.176^{\circ }$	${{\bf 0.425}^{\circ }}$	$0.313^{\circ }$	$0.093^{\circ }$
MAE loss	$\underline{0.194^{\circ }}$	$0.437^{\circ }$	$\underline{0.294^{\circ }}$	${{\bf 0.056}^{\circ }}$
Huber loss	${{\bf 0.128}^{\circ }}$	$\underline{0.432^{\circ }}$	${{\bf 0.282}^{\circ }}$	$\underline{0.065^{\circ }}$

## Conclusion

In this work, we present a novel U-shaped network structure for spacecraft ISAR imaging pose estimation. Our approach addresses the challenges of large noise, fuzzy boundaries, and limited data in spacecraft ISAR imaging. To improve image quality, we propose the use of CLAHE for noise reduction and contrast enhancement. Additionally, we leverage pre-trained models to mitigate the impact of dataset size on model performance. To overcome sparsity and feature loss during transmission, we introduce a dense residual block and a multi-head attention block. These blocks enable our model to capture more features, enhance feature transmission, and recover lost information. Experimental results demonstrate that our approach outperforms both human pose estimation and existing spacecraft pose estimation methods using the speedplus dataset. In the future, we aim to further optimize our model for lightweight spacecraft pose estimation by reducing the number of parameters.

## Data Availability

The datasets generated and/or analyzed during the current study are not publicly available due to confidentiality but are available from the corresponding author upon reasonable request.
